# Supplementary material for: Mechanisms governing the pioneering and redistribution capabilities of the non-classical pioneer PU.1
Source: Nat Commun. 2020 Jan 21;11:402. doi: 10.1038/s41467-019-13960-2 (PMC6972792; doi:10.1038/s41467-019-13960-2)
Supplement: Supplementary file 7 — Source data [file 41467_2019_13960_MOESM7_ESM.zip › Source_Data/FigureS5/FigureS5A_MotifsCommonPeaks/homerResults/motif9.similar.html]

motif9

## Information for motif9

T
C
G
A
G
C
T
A
T
A
C
G
G
C
T
A
A
C
T
G
T
C
A
G
G
T
C
A
G
C
T
A
T
A
C
G
C
G
T
A
A
C
T
G
T
C
A
G
G
T
C
A
C
T
G
A
  
Reverse Opposite:  

G
A
C
T
A
C
G
T
A
G
T
C
T
G
A
C
G
C
A
T
A
T
G
C
C
G
A
T
C
A
G
T
A
G
T
C
T
G
A
C
C
G
A
T
A
T
G
C
C
G
A
T
A
G
C
T
  

|  |  |
| --- | --- |
| p-value: | 1e-165 |
| log p-value: | -3.816e+02 |
| Information Content per bp: | 1.647 |
| Number of Target Sequences with motif | 938.0 |
| Percentage of Target Sequences with motif | 10.56% |
| Number of Background Sequences with motif | 1173.6 |
| Percentage of Background Sequences with motif | 3.09% |
| Average Position of motif in Targets | 97.2 +/- 54.2bp |
| Average Position of motif in Background | 112.1 +/- 72.5bp |
| Strand Bias (log2 ratio + to - strand density) | -0.3 |
| Multiplicity (# of sites on avg that occur together) | 1.01 |
| Motif File: | file (matrix) reverse opposite |

### Similar de novo motifs found

|  |  |  |  |  |  |  |  |
| --- | --- | --- | --- | --- | --- | --- | --- |
| Rank | Match Score | Redundant Motif | P-value | log P-value | % of Targets | % of Background | Motif file |
| 1 | 0.807 | T C G A T A G C T C G A C A T G T C A G T G C A G T C A A T C G C T G A A C T G T A C G T C G A C T G A | 1e-149 | -344.812936 | 9.66% | 2.84% | motif file (matrix) |
| 2 | 0.765 | C G T A A C T G A C T G C G T A C G T A A C T G C G T A C G T A A C T G C G T A A C T G A C T G C G T A C G T A | 1e-92 | -213.543790 | 6.79% | 2.19% | motif file (matrix) |
